# Sex differences in neurocognitive performance in older adults with bipolar disorder

**DOI:** 10.1192/j.eurpsy.2024.912

**Published:** 2024-08-27

**Authors:** S. Martín-Parra, C. Torrent, A. Ruiz, M. Bort, G. Fico, V. Oliva, M. D. Prisco, J. Sanchez-Moreno, E. Jimenez, A. Martinez-Aran, E. Vieta, B. Sole, L. Montejo

**Affiliations:** ^1^Bipolar and Depressive Disorders Unit, Hospital Clinic of Barcelona, IDIBAPS, University of Barcelona; ^2^Bipolar and Depressive Disorders Unit, Hospital Clinic of Barcelona, CIBERSAM, IDIBAPS, University of Barcelona; ^3^Bipolar and Depressive Disorders Unit, Hospital Clinic of Barcelona, CIBERSAM, IDIBAPS, Departament de Medicina, Facultat de Medicina i Ciències de la Salut, University of Barcelona, Barcelona, Spain

## Abstract

**Introduction:**

In recent years, research has focused on the older adults with bipolar disorder (OABD), aged 50 years and over, a constantly growing population due to the increased of life expectancy. Actually, some authors suggest that these individuals constitute a distinct subtype with a specific and different needs such as seen in epidemiologic, clinical and cognitive features. Further research has revealed significant differences between females and males with BD in clinical and cognitive variables in middle-aged and young patients, but this topic among OABD population remains unclear.

**Objectives:**

The aim of this study is to identify the distinctive profile in clinical, functional and neurocognitive variables between females and males in OABD.

**Methods:**

A sample of OABD and Healthy Controls (HC) were included. Euthymic patients or in partial remission were included. Neurocognition was measured with a battery of tests that included premorbid intelligence quotient, working memory, verbal and visual memory, processing speed, language and executive functions. Independent t-test and Chi-squared test analysis were performed as appropriated.

**Results:**

According to the analysis, statistically significant differences were seen between females and males. A more impaired cognitive profile is observed in women. They performed worse in the subscales of Arithmetic (F= 6.728, *p* = <0.001), forward digits (F= 0.936, *p*= 0.019) and Total Digits (F= 1.208, *p*= 0.019) of the WAIS-III, in the Stroop Color Word Test, color reading (F= 0.130, *p*= < 0.001), in the Continuous Performance Test, block change measure (F= 2.059, *p*= 0.037), in the Rey-Osterrieth Complex Figure-copy (F= 0.005, *p*= 0.029) and in the Boston Naming Test (F= 0.011, *p*= 0.024). Nor significant differences were found in clinical neither in psychosocial functioning variables.

**Conclusions:**

In view of the following results, and since no differences were observed between women and men in terms of clinical and functional outcomes, it could be said that the differences observed in cognition cannot be explained by disease-related factors. Furthermore, these results highlight the need to develop a gender-specific cognitive interventions in OABD population. In this way, we could have an impact on the course of the illness to reach a better quality of life.

**Disclosure of Interest:**

S. Martín-Parra: None Declared, C. Torrent Grant / Research support from: Spanish Ministry of Science and Innovation (PI20/00344) integrated into the Plan Nacional de I+D+I and co-financed by the ISCIIISubdireccion General de Evaluación and the Fondo Europeo de Desarrollo Regional (FEDER), A. Ruiz: None Declared, M. Bort: None Declared, G. Fico Grant / Research support from: Fellowship from “La Caixa” Foundation (ID 100010434 - fellowship code LCF/BQ/DR21/11880019), V. Oliva: None Declared, M. Prisco: None Declared, J. Sanchez-Moreno Grant / Research support from: Spanish Ministry of Science and Innovation (PI20/00060) integrated into the Plan Nacional de I+D+I and co-financed by the ISCIII-Subdireccion General de Evaluación and the Fondo Europeo de Desarrollo Regional (FEDER), E. Jimenez Grant / Research support from: Spanish Ministry of Science and Innovation (PI20/00060) integrated into the Plan Nacional de I+D+I and co-financed by the ISCIII-Subdireccion General de Evaluación and the Fondo Europeo de Desarrollo Regional (FEDER), A. Martinez-Aran: None Declared, E. Vieta Grant / Research support from: Spanish Ministry of Science and Innovation (PI18/ 00805, PI21/00787) integrated into the Plan Nacional de I+D+I and cofinanced by the ISCIII Subdirección General de Evaluación and the Fondo Europeo de Desarrollo Regional (FEDER); the Instituto de Salud Carlos III; the CIBER of Mental Health (CIBERSAM); the Secretaria d’Universitats i Recerca del Departament d’Economia i Coneixement (2017 SGR 1365), the CERCA Programme, and the Departament de Salut de la Generalitat de Catalunya for the PERIS grant SLT006/17/00357; the European Union Horizon 2020 research and innovation program (EU.3.1.1. Understanding health, wellbeing and disease: Grant No 754907 and EU.3.1.3. Treating and managing disease: Grant No 945151), B. Sole: None Declared, L. Montejo: None Declared

